# Smoking Is Associated With Low Levels of Soluble PD-L1 in Rheumatoid Arthritis

**DOI:** 10.3389/fimmu.2018.01677

**Published:** 2018-07-27

**Authors:** Caroline Wasén, Malin C. Erlandsson, Apostolos Bossios, Linda Ekerljung, Carina Malmhäll, Sofia Töyrä Silfverswärd, Rille Pullerits, Bo Lundbäck, Maria I. Bokarewa

**Affiliations:** ^1^Department of Rheumatology and Inflammation Research, Institute of Medicine, Sahlgrenska Academy, University of Gothenburg, Gothenburg, Sweden; ^2^Krefting Research Centre, Department of Internal Medicine and Clinical Nutrition, Institute of Medicine, University of Gothenburg, Göteborg, Sweden; ^3^Department of Clinical Immunology and Transfusion Medicine, Sahlgrenska University Hospital, Gothenburg, Sweden

**Keywords:** rheumatoid arthritis, programmed death protein 1, soluble programmed death protein 1, smoking, Fc-gamma receptors, autoantibodies, TNF-a inhibitors

## Abstract

**Background:**

Smoking is a risk factor for developing rheumatoid arthritis (RA), but the mechanism remains uncertain. We previously demonstrated that smoking lowers the T cell activation threshold by limiting programmed death protein 1 (PD-1) expression.

**Aim:**

To investigate how smoking influence the levels of soluble PD-1 ligand (sPD-L1).

**Method:**

Serum levels of sPD-L1 were measured in 246 RA patients and in 168 healthy subjects. The analysis was done with respect to inflammation, smoking, treatments, and autoantibody status. The effect of therapeutic TNF-inhibiting antibodies (TNFi) on sPD-L1 was studied in 16 RA patients at their first infliximab infusion. The expression of Fcγ-receptor (FcγR) subclass IIB and IIIA was analyzed with quantitative polymerase chain reaction in peripheral blood mononuclear cells (PBMCs) from 12 RA patients and 15 healthy controls, and in healthy PBMC exposed to IgG containing antibodies to cyclic citrullinated peptides (aCCP).

**Results:**

The negative association between smoking and sPD-L1 in RA patients was established by multiple logistic regression (OR = 0.52, *p* = 0.038). Other covariates in the regression model were serum levels of IL-1β representing inflammation (OR = 1.6, *p* = 0.0076) and aCCP positivity (OR = 1.9, *p* = 0.047). First infliximab infusion repressed sPD-L1 (*p* = 0.023) in patients, and low levels of sPD-L1 were found in patients with early RA treated with TNFi (*p* = 0.018). Treatment with TNFi was associated with higher sPD-L1 in patients with long disease duration (*p* = 0.041) and restored levels in smokers. *In vitro* exposure to aCCP+ IgG suppressed sPD-L1 (*p* = 0.036), but aCCP+ patients with long disease duration had higher sPD-L1 (*p* = 0.016). High ratio of the inhibitory FcγR subclass IIB over the stimulatory IIIA resulted in low sPD-L1 release (*p* = 0.029). Smoking was associated with a higher FcγR IIB/IIIA ratio (*p* = 0.00062) and lower levels of sPD-L1 (*p* = 0.013).

**Conclusion:**

In RA, serum sPD-L1 was related to systemic inflammation and aCCP positivity. Smoking altered the expression of FcγRs and limited sPD-L1 in RA patients, permitting inappropriate T cell responses. Differential regulation of sPD-L1 during the early and late RA may indicate transposition from acute to chronic inflammation.

## Introduction

Within the past 5 years, the interest in the T cell co-receptor programmed death protein 1 (PD-1) has grown exponentially. Much of this interest is explained by the success of the first therapeutic antibody targeting PD-1 that was approved by The Food and Drug Administration as a new immunotherapy for malignant melanoma in 2014 (1). T cells play a crucial role in the specific immune defense against both infections and malignancies. They kill defective cells either directly or by activating appropriate B cells. Under physiological conditions, their cytotoxic activity is tightly regulated to prevent tissue damage and autoimmunity. PD-1 together with its ligands PD-L1 and PD-L2 presents a major regulatory system controlling cytotoxicity (2). Activation of PD-1 by its ligands inhibits the stimulatory signal from the T cell receptor, increasing its threshold of activation. In addition, PD-L1 is a ligand for CD80 that is also involved in negative co-stimulation of T cells (3). In cancer, tumor cells commonly evade T cell-mediated destruction by up-regulating PD-L1, but this inhibitory interaction can be counteracted by the administration of PD-1 blocking antibodies. However, the autoimmune situations developed as a result of PD-1 inhibition clearly demonstrate the importance of this defense mechanism against autoimmunity (4).

Rheumatoid arthritis (RA) is an autoimmune disease characterized by chronic inflammation of the joints. Several cases of RA flare have been reported among patients treated with PD-1 blocking antibodies (5), indicating that RA can be triggered by a defective PD-1/PD-L1/2 inhibitory system. The etiology of RA is to a large extent uncertain which obstruct the development of new effective treatments. Several risk factors have been associated with RA development including a combination of genetic and environmental components (6). The environmental factor that has been given the most attention in the etiology of RA is smoking. The increased risk to develop RA depended primarily on the exposure to cigarette smoke (7). In heavy smokers, the RA risk was increased as long as 20 years after smoke cessation. We have previously demonstrated that smoking limited the expression of PD-1 by CD8+ T cells in RA patients and in healthy subjects, suggestively through the direct stimulation of nicotinic receptors (8).

With the diverse etiology of RA in mind, it is not surprising that the pathogenesis of RA vary between individuals. A common approach to separate patients with different pathogenic ways is by the presence of autoantibodies rheumatoid factor and anti-citrullinated peptide antibodies (aCCP) in serum (9). The positivity for autoantibodies is typically related to a worse RA prognosis. Of note, RA as a result of PD-1 blockade has repeatedly been presented with positivity for both autoantibodies (5).

Several reports indicate an association between PD-L1 and RA pathology. Dendritic cells and monocytes typically express PD-L1, but non-hematopoietic cells and malignant cells may also express PD-L1 (10). In endothelial cells, macrophages, and dendritic cells, expression of PD-L1 is induced by inflammatory stimuli through type I and type II interferon signaling (11, 12). In human cancer cells, activation of the JAK/STAT pathway leads to the binding of IFN regulatory factor-1 to the PD-L1 gene promoter (13), while activation of the MEK/ERK pathway leads to the binding of STAT1 transcription factor to the PD-L1 gene promoter (14). Other potent inducer of PD-L1 is tumor necrosis factor α (TNF-α), functional both in monocytes (15) and in cancer cells (16). The soluble form of PD-L1 (sPD-L1) is thought to be cleaved off the membrane by matrix metalloproteinases (17). Previous reports suggested that serum levels of sPD-L1 in RA reflected the activity of PD-L1 producing CD14+ monocytes in peripheral blood (18). In addition, the levels of PD-L1 in synovial fluid were high due to its high expression by the inflamed RA synovial tissue (19). Importantly, PD-L1 seems to have a protective effect in arthritis. In experimental arthritis, PD-1 knockout aggravated arthritis, while ligation of PD-1 using a soluble murine PD-L1-Fc-fusion protein ameliorated the disease (19). Collagen-induced arthritis has been successfully treated with PDL-Ig, decreasing splenocyte proliferation and production of IL-17 and IL-23 (20). Similarly, administration of a PD-L1-Fc-fusion protein decreased IL-17 production in mice with imiquimod-induced psoriatic inflammation (21).

In the present report, we further explore the effect of smoking on the PD-1/PD-L1 inhibitory system in RA with focus on sPD-L1. For this purpose, we analyze the sPD-L1 levels in serum of RA patients and healthy subjects with known smoking status in relation to the expression of FcγRs in leukocytes of the peripheral blood. We also investigate the role of therapeutic TNFα-inhibiting antibodies and RA-specific aCCP antibodies in the regulation of sPD-L1 production.

## Materials and Methods

### Patients and Healthy Controls

A total of 246 RA patients visiting the Rheumatology units at the Sahlgrenska University Hospital in Gothenburg and the Northern Älvsborg County Hospital in Uddevalla, Sweden were randomly recruited from the methotrexate (MTX) registers to participate in the study between 2011 and 2013. The demographic characteristics and anti-rheumatic treatment of the study participants at enrollment are summarized in Table 1. All the RA patients fulfilled the 1987 American College of Rheumatology classification criteria for RA (22). Rheumatologists examined patients when they were included in the study.

**Table 1:** Demographic and clinical characteristics of RA patients and healthy controls.

	Patients	Healthy controls
Total number	246	168
Female, *n* (%)	180 (73)	104 (56)
Age, range of years	21−71	40−77
Nicotine users within 25 years, *n* (%)	106 (57)	96 (57)
Disease duration, range of years	1−49	NA
**Autoantibodies, *n* (%)**		
Anti-cyclic citrullinated peptide positive	145 (60)	NA
RF positive	175 (82)	NA
**Treatments, *n* (%)**		NA
Prednisolone	29 (12)	
Methotrexate	220 (89)	
Anti-CD20 antibodies	12 (5)	
Anti-IL-6R antibodies	7 (3)	
CTLR4-fusion protein	2 (1)	
TNFα-inhibitors	73 (31)	

Clinical activity of RA was calculated at the time of blood sampling based on the number of swollen and tender joints, erythrocyte sedimentation rate (ESR) and global health assessment of the patient, and the disease activity score (DAS28) was constructed (23). DAS28 above 3.2 indicated the presence of active RA disease.

All patients gave written informed consent. The study was approved by the Regional Ethical Evaluation Board in Gothenburg, Sweden. Trail registration: ClinicalTrials.gov NCT03449589.

Serum samples of 168 healthy controls were randomly selected from the healthy participants of the West Sweden Asthma Study (24) to match the cross-sectional RA cohort with regards to age and gender. The West Sweden Asthma study is a large-scale epidemiological evaluation of the prevalence of asthma and respiratory symptoms in adults between the ages of 16 and 75 in West Sweden including a group of study participants that underwent a clinical examination and blood sampling.

Information about smoking habits, medication, and concomitant diseases were collected using a structured questionnaire at visit from RA patients and through a structured interview of the healthy subjects. We considered the current smokers and the individuals who smoked within the past 25 years as smokers, while never-smokers and individuals who stopped smoking longer than 25 years ago were regarded as non-smokers. The use of moist snuff alone or in combination with smoking was also considered smoking.

### Infliximab Treatment

In total 16 RA patients, 13 females and 3 males with disease duration of 1–32 years, participated in the pilot study of short-term serological effects of infliximab. Clinical characteristics of the patients are shown in Figure 3C. At enrollment, all the patients were naïve to any therapeutic TNFα-inhibitors. The pre-infusion blood samples were collected the morning before the first infusion of infliximab (Remicade; Schering-Plough, Kenilworth, NJ, USA). Infliximab treatment was provided intravenously in a dose of 200 mg at the Rheumatology Clinics, Sahlgrenska University Hospital. For the second blood sampling, 8 patients returned 24 h after the infliximab infusion, 7 patients returned after 2 weeks. In 7 patients the blood sample was taken after 6 weeks. These patients received two infusions of infliximab.

### Blood Sample Preparation and Storage

Blood samples were obtained from the cubital vein using the vacutainer (Greiner bio-one, Kremsmünster, Austria). All blood samples were centrifuged at 800 × *g* for 15 min, aliquoted, and stored frozen at −70°C until use.

### Immunoglobulin G (IgG) Isolation

IgG was isolated from serum samples of 3 aCCP-negative and 4 aCCP-positive RA patients. All the aCCP-positive patients were also positive for RF. Isolation was performed using HiTrap Protein A HP (GE Healthcare, Marlborough, MA, USA) according to manufacturer’s protocol. The IgG was eluted in 5 ml 0.1 M citric acid and the pH was neutralized with 180 µl Tris–HCl pH 9 per ml of the eluted citric acid. The IgG was further concentrated using Vivaspin 6 with 50,000 MWCO PES membranes (Satorius AG, Göttingen, Germany). Concentration of IgG was determined using NanoDrop spectrophotometer (ThermoFisher, Waltham, MA, USA). 100 µl of aCCP positive IgG (10 µg/ml in PBS) was then used to coat the bottom of a 96-well cell culture plate in room temperature over night. We used a direct enzyme-linked immunosorbent assay (ELISA) to confirm adherence of IgG to the plate bottom (see below).

### Cell Isolation and Culture

For *in vitro* stimulation, we collected blood samples from four healthy women (age 45–54 years). Mononuclear cells were isolated immediately after the blood sampling through gradient centrifugation on Lymphoprep (Fresenius Kabi, Oslo, Norway). Cells were washed twice in PBS and suspended in RPMI Medium 1640 + GlutaMAX-1 culture media (Gibco by Life technologies, Grand Island, NY, USA) supplemented with 10% fetal calf serum (SigmaAldrich, St. Louis, MO, USA), 50 µg/ml gentamicin (Sanofi-Aventis, Paris, France) and 50 µM β-mercaptoethanol (Gibco by Life technologies). Cells were seeded 1 × 10^9^/ml on the IgG-coated plates and cultured at 37°C with 5% CO_2_ in the presence or absence of 10 µg/ml lipopolysaccharide (LPS, Sigma). After 72 h, the cell supernatant was collected and cells were lysed in RLT/β_2_-ME buffer (Qiagen, Hilden, Germany).

For transcriptional analysis of FcγR expression, blood samples were collected from 15 female RA patients (age 42–76 years, 7 smokers) and 12 healthy females (age 43–80 years, 4 smokers). Mononuclear cells were isolated from the peripheral blood immediately after the blood sampling through gradient centrifugation on Lymphoprep (Fresenius Kabi). Antigen-presenting cells were prepared by the consequent depletion of CD4+ cells using Dynabeads CD4 positive isolation kit (Life technologies, Oslo, Norway) and CD8+ cells using Human CD8+ T cell enrichment kit (Stemcell Technologies, Vancouver, BC, USA). The sorted cells were counted, the concentration was adjusted to 1–2 × 10^6^/ml in RPMI and activated for 1 h at 37°C, 5% CO_2_ with phorbol 12-myristate 13-acetate (PMA, 30 ng/mL; Sigma-Aldrich), and ionomycin (0.5 µg/mL; Sigma-Aldrich) as described (25). The supernatants were discharged and the cells were lysed in 350 µl RLT/β_2_-ME buffer for quantitative polymerase chain reaction (qPCR) analysis.

### Enzyme-Linked Immunosorbent Assay

Serum levels of sPD-L1 were analyzed in the samples diluted 1:2 in PBS supplemented with 1% bovine serum albumin (1% BSA–PBS) using human PD-L1/B7-H1 DuoSet ELISA (DY156, R&D Systems). The protocol was optimized with respect to antibody concentration. The mouse anti-human B7-H1 capture antibody was diluted 1:90 and the biotinylated goat anti-human B7-H1 detection antibody was diluted 1:60 in 1% BSA–PBS. Streptavidin conjugated horseradish peroxidase and 3,3′5,5′-tetramethylbenzidine (TMB) was used for development. The absorbance was read at 450/650 nm dual wavelengths with SPECTRAmax 340PC384 microplate reader and SoftMax Pro software (Molecular Devices, San Jose, CA, USA). The standard curve prepared with human recombinant B7-H1 and ranging from 0.3 to 2500 pg/ml was used for calculation of the absolute PD-L1 levels in the samples.

Serum levels of interleukins (IL) IL-6 and IL-1β were analyzed in 1:2 dilutions with PeliPair reagent set (Sanquin, Amsterdam, the Netherlands) according to the manufacturer’s instructions. The minimal detection level for IL-6 and IL-1b was 0.2 pg/ml.

The levels of aCCP antibodies were measured by the automatic multiplex method using the anti-CCP2 kit (BioRad, Hercules, CA, USA). The cut-off level above 3.0 U/mL was set positive by the manufacturers and verified on healthy individuals. For cell stimulation, aCCP content of serum was determined by the Immunoscan CCPlus kit according to the manufacturer’s instruction (Euro Diagnostica, Malmö, Sweden).

The coating of cell culture plate with human IgG was confirmed by direct ELISA using a biotinylated *F*(ab′)2 goat anti-human IgG detection antibody (Jackson Immunoresearch, West Baltimore Pike, West Grove, PA, USA).

### Quantitative Polymerase Chain Reaction

The total RNA was prepared from the cell lysates using RNeasy Mini Kit (Qiagen). RNA concentration was measured using NanoDrop. Complementary (c)DNA was then synthesized using the High Capacity cDNA Reverse Transcription Kit (Applied Biosystems, Foster City, CA, USA). The gene-specific primers were designed^1^^,^2^,^3^,^4^^ to generate a product spanning two exons to avoid unspecific signal from genomic DNA (Table 2) and synthesized by Sigma-Aldrich. Amplification of the gene product was performed using SYBR Green qPCR Mastermix (SABiosciences, Qiagen) and ViiA™ 7 Real-Time PCR (Applied Biosystems). The quality of the results was analyzed using melting curves obtained between 60 and 95°C. The relative gene expression was calculated using the ddCt-method with glyceraldehyde-3-phosphate dehydrogenase as reference gene.

**Table 2:** Primer sequences (5′–3′).

Primer	Forward primer	Reverse primer
FCGR3A	CCTCCCAACTGCTCTGCTAC	TCTCGAGCACCCTGTACCAT
FCGR2B	ACGCTGTACTCATCCAAGCC	CCCCAACTTTGTCAGCCTCA
PD-L1	GCTATGGTGGTGCCGACTAC	GGACTTGATGGTCACTGCTTG

### Statistics

GraphPad Prism v.7 for Mac OS X (GraphPad Software, La Jolla, CA, USA, www.graphpad.com) was used for graphical representation of the data. Data were plotted as the median with interquartile range. The Mann–Whitney *U*-test or the Wilcoxon matched-pair signed rank test was used to compare the differences between the groups. For group-wise comparison, the patients were stratified based on smoking status, and further sub-divided based on treatment with TNFα-inhibiting antibodies. Patients were also stratified for aCCP and disease duration. The Spearman’s rank correlation test was used to calculate *r*- and *p*-values of correlating data. A *p*-value below 0.05 was considered statistically significant.

The association between the dependent variable, sPD-L1, and the independent variables; smoking; IL-6, IL-1β, and ESR (systemic inflammation); DAS28 (disease activity) and disease duration, aCCP and RF (autoantibodies); MTX and TNF-α inhibiting therapeutic antibodies (TNFi, treatments); sex and age was tested using the Spearman’s correlation, to establish associations, and the Pearson’s correlation, to test linearity. Correlation plots were generated in R version 3.4.1 (26). Since sPD-L1 levels were not normally distributed, the association between sPD-L1 levels and smoking was tested by logistic regression using IBM SPSS Statistics Version 24.0 (IBM corp.). For this purpose sPD-L1 was dichotomized. The patients were arbitrarily divided at the 0.4 quantile. Patients with more than 3 pg/ml were assigned the value of 1 and the rest 0. The distribution of sPD-L1, IL-6, and IL-1β levels was positively skewed and thus log10 transformed to achieve a linear relationship between sPD-L1 and the independent variables. Smokers, males, aCCP+, RF+, and TNFi-treated patients were assigned 1 and the rest 0. All variables with a significant correlation (*p* < 0.05) to sPD-L1 levels were entered to the regression model that assessed their contribution to the model by the backward Wald method. Only complete cases were analyzed.

## Results

### Soluble PD-L1 Is Connected to Inflammation and to Presence of Autoantibodies in RA Patients

**Figure 1:**
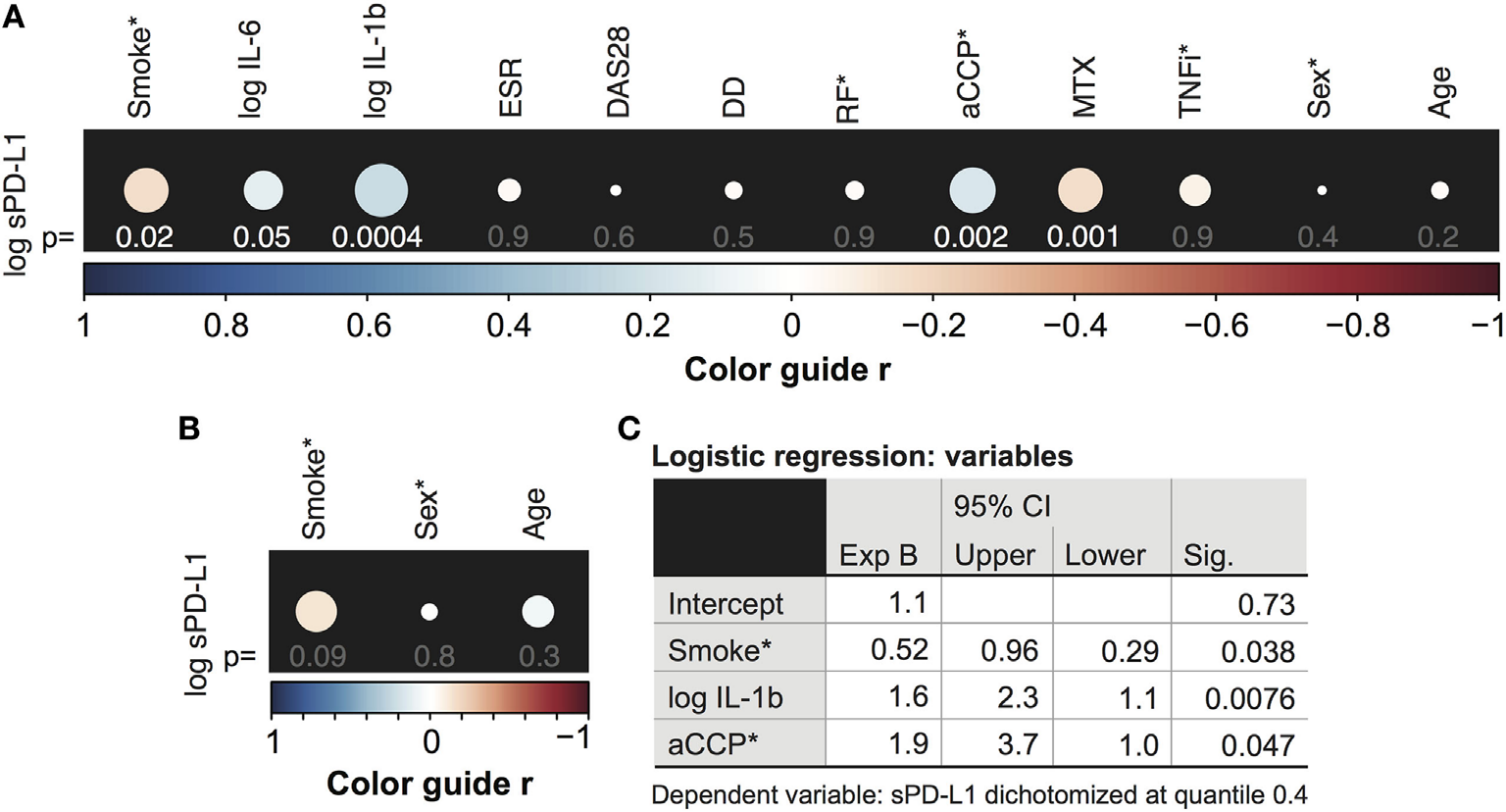
Serum levels of soluble PD-L1 (sPD-L1) are low in smokers. **(A,B)** Spearman correlation between the logarithm of sPD-L1 and interleukins (IL)-6 and IL-1β, disease activity score (DAS28), erythrocyte sedimentation rate (ESR), disease duration (DD), anti-cyclic citrullinated peptide (aCCP) and rheumatoid factor (RF), weekly dose of methotrexate (MTX), anti-tumor necrosis factor α antibody (TNFi) treatment **(A)**, and age and sex **(A,B)**. sPD-L1 was measured in 246 RA patients **(A)** and 168 healthy controls **(B)**. The color guide shows the value of *r*. **(C)** Results of the logistic regression analysis. sPD-L1 was dichotomized into groups with low levels (0) and high levels (1). The variables indicated with (*) are nominal or ordinal and only take on values 1 and 0. Smokers, aCCP+, RF+, and TNF-inhibiting antibodies treated and males are assigned number 1.

Our initial aim was to determine the relationship between the sPD-L1 production and inflammation in a cohort of 246 RA patients. Thus, an explorative analysis of associations between sPD-L1 and several inflammation and disease parameters was conducted. The results are presented in Figure 1A. Inflammation parameters included serum levels of IL-6, IL-1β, and ESR. RA characteristics were DAS28, disease duration (DD), and autoantibodies aCCP and RF, in addition to immunosuppressive treatments MTX and TNFi. The demographic parameters, age and sex were used in both RA patients and 168 healthy controls.

There was a significant correlation between the logarithm of sPD-L1 and serum levels of IL-6 [Figure 1A, *r* = 0.13, 95% confidence interval (CI) = −0.0023 to 0.25, and *p* = 0.048] and IL-1β (*r* = 0.22, 95% CI = 0.098–0.34, and *p* = 0.00041, Figure 1A). However, there was no association of sPD-L1 with DAS28, ESR, or DD. The aCCP status was positively correlated with sPD-L1 (*r* = 0.21, 95% CI = 0.076–0.34, and *p* = 0.0020), but RF was not correlated. The weekly dose of MTX displayed a negative correlation with the sPD-L1 levels (*r* = −0.21, 95% CI = −0.33 to −0.079, and *p* = 0.0014), but there was no correlation with TNFi treatment. There was no association between log sPD-L1 and age or sex among RA patients and healthy controls.

### Soluble PD-L1 Levels Are Low in Smokers but Stimulated by Autoantibodies

We have previously reported that smoking interferes with PD-1/PD-L1-mediated regulation of T cells by limiting the expression of PD-1 (8). To investigate if smoking also influences PD-L1 we analyze serum levels of sPD-L1 in RA patients and healthy controls. The initial analysis revealed a negative correlation between sPD-L1 and the smoking status of RA patients (Figure 1A, *r* = −0.15, 95% CI = −0.28 to −0.026, and *p* = 0.016). This correlation was not significant in healthy controls (Figure 1B, *r* = −0.14, 95% CI = −0.29 to 0.021, and *p* = 0.090).

The independent association between smoking and sPD-L1 levels was analyzed by multiple logistic regression analysis, to control the confounders identified by univariate logistic regression analysis. The variables tested by multivariate analysis were smoking (*p* = 0.012), the logarithm of IL-1β (*p* = 0.013), the weekly dose of MTX (*p* = 0.011), and the aCCP status (*p* = 0.0040). Patients were stratified into two groups based on their sPD-L1 levels. Because 37% of the patients had values below the level of detection, we set the limit between low and high sPD-L1 level at the 0.4 quantile. The analysis revealed that smokers were unlikely of having high sPD-L1 levels [Figure 1C, odds ratio (OR) 0.52, 95% CI = 0.29–0.96, and *p* = 0.038], meaning that smoking was significantly associated with low sPD-L1 levels when potential confounders were accounted for. Other covariates that significantly contributed to the prediction of sPD-L1 were IL-1β (OR 1.6, 95% CI = 1.1–2.3, and *p* = 0.0080) and aCCP (OR 1.9, 95% CI = 1.0–3.7, and *p* = 0.047).

### Anti-CCP Antibodies Influence sPD-L1 Levels Differently Depending on Time of Exposure

**Figure 2:**
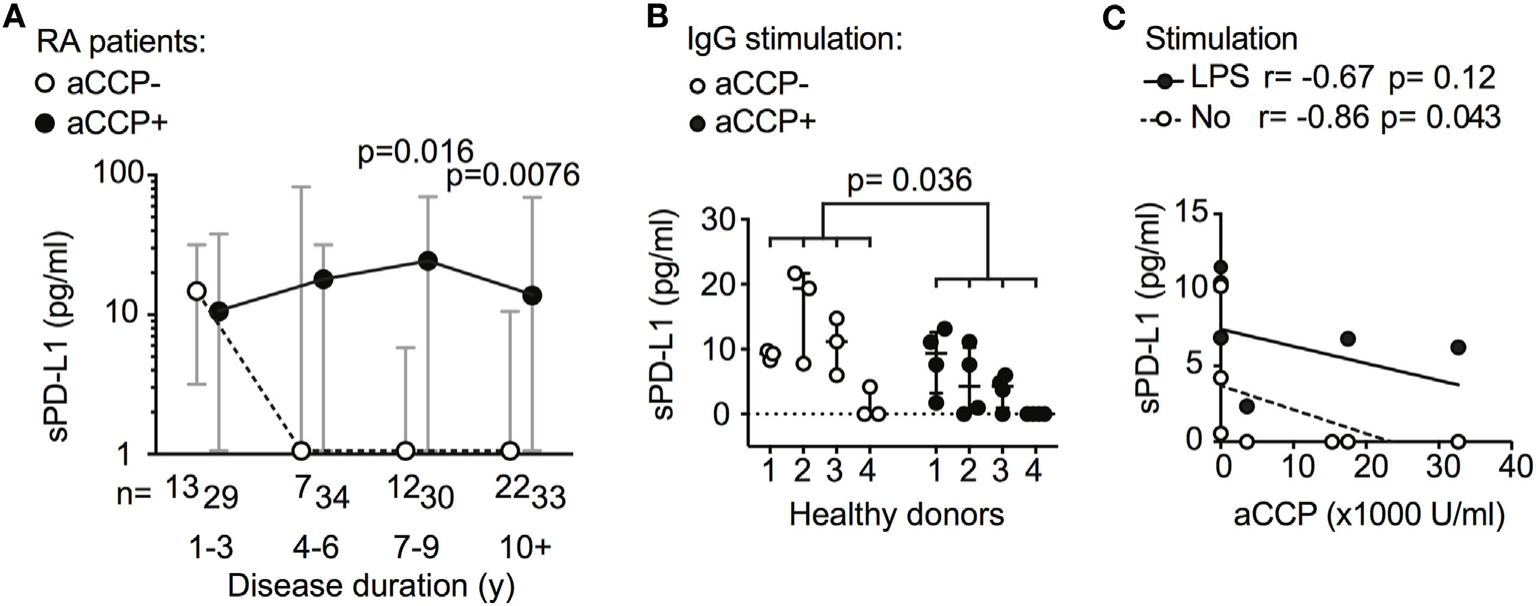
Antibodies against anti-cyclic citrullinated peptides (aCCP) influence production of soluble PD-L1 (sPD-L1). **(A)** sPD-L1 in serum of aCCP+ and aCCP− RA patients, stratified by disease duration. Dots represent the median and whiskers represent the interquartile range. **(B)** sPD-L1 measured in lipopolysaccharide (LPS) stimulated cultures of peripheral blood monocytes from aCCP− healthy controls exposed to IgG of aCCP+ and aCCP− RA patients. The Mann–Whitney *U* test was applied. **(C)** Spearman’s rank correlation test between sPD-L1 in supernatant of healthy peripheral blood mononuclear cells cultures and aCCP content in serum of the patients from which the IgG was isolated. Cells were cultured with or without LPS stimulation. Circles represent median sPD-L1 level of each IgG sample (aCCP− *n* = 3, aCCP+ *n* = 4).

Further exploring the association between sPD-L1 and aCCP status, we subdivided the RA patients by their disease duration. The disease duration served as a rough approximation of the exposure time to aCCP. In early RA (DD < 3 years), aCCP-positive and aCCP-negative patients had similar levels of sPD-L1. In established RA (DD > 7 years), aCCP-positive patients had significantly higher levels of sPD-L1 (Figure 2A, median 1.0 vs. 23 pg/ml, Hodges–Lehmann (H–L) difference = 20, 95% CI = 0.0–39, and *p* = 0.016). sPD-L1 levels remained high in patients with DD > 10 years (median 1.0 vs. 13 pg/ml, H–L difference = 5.0, 95% CI = 0.0–23, and *p* = 0.0076).

To study the immediate effect of aCCP on the production of sPD-L1, we conducted an *in vitro* experiment. PBMCs from four healthy women were exposed to aCCP-positive or aCCP-negative IgG isolated from serum of RA patients. In the presence of LPS, the cells exposed to aCCP-positive IgG produced lower levels of sPD-L1 (Figure 2B, median 8.8 vs. 2.7 pg/ml, H–L difference = 5.1, 95% CI = −9.3 to 0.0, and *p* = 0.036). The production of sPD-L1 was negatively correlated with the aCCP concentration (Figure 2C, *r* = −0.86, and *p* = 0.043).

### Therapeutic Antibodies Suppress sPD-L1 Production After Short Exposure

**Figure 3:**
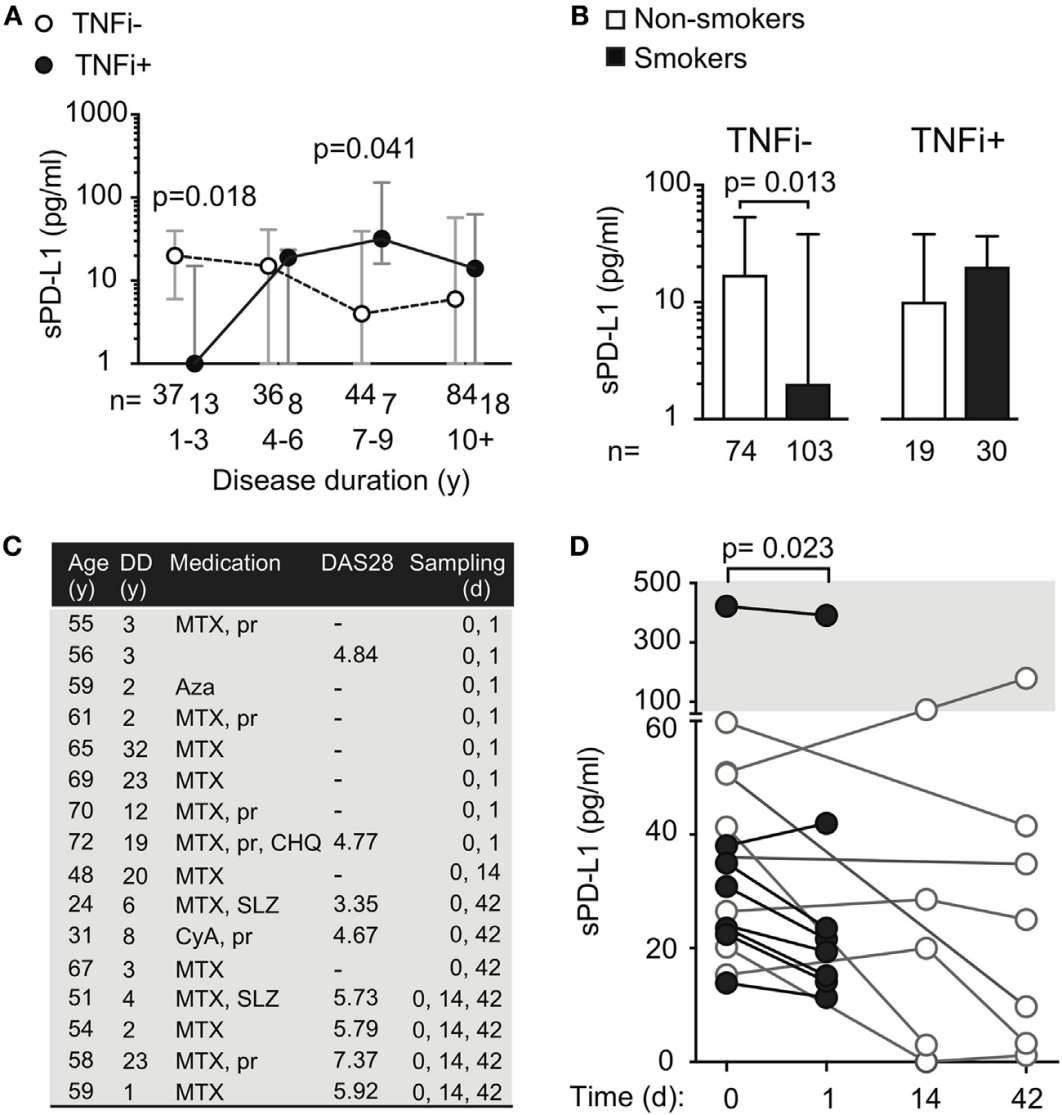
Treatment with tumor necrosis factor α inhibiting antibody (TNFi) influences serum levels of soluble PD-L1. **(A)** Soluble PD-L1 (sPD-L1) in serum of RA patients treated and not treated with TNFi, stratified by disease duration. Dots represent the median and whiskers represent the interquartile range. **(B)** sPD-L1 in RA patients who are smokers or non-smokers, stratified by TNFi treatment. The Mann–Whitney *U* test was applied. **(C,D)** sPD-L1 in RA patients before, after 1 day, 2 weeks, and 6 weeks of the first infliximab infusion of infliximab. Clinical parameters are described in **(C)**. The Wilcoxon matched-pairs signed rank test was applied. **(C)** Patient information. Abbreviations: MTX, methotrexate; pr, prednisolone; Aza, azathioprine; CHO, chlorokinolone; SLZ, sulfasalazine; CyA, cyclosporine A.

We asked if RA-specific antibodies were unique in their ability to induce sPD-L1 in RA patients. To address this, we compared sPD-L1 levels in RA patients with different disease duration by stratifying patients on TNFi treatment status. The studied RA cohort contained 50 patients treated with TNFi. Among them, 38 patients were treated with infliximab, 5 patients with adalimumab, and 7 patients were treated with golimumab. Those 20 patients who were treated with etanercept, a Fc-fusion protein interacting with TNFα receptor, were excluded from the analysis. We observed that patients with DD < 3 years treated with TNFi had significantly lower sPD-L1 (Figure 3A, median 0.0 vs. 20 pg/ml, H–L difference = −12, 95% CI = −28 to 0.0, and *p* = 0.018). However, this difference was reversed in patients with DD of 7–9 years. Here, TNFi-treated patients had higher sPD-L1 (median 4 vs. 32 pg/ml, H–L difference = 20, 95% CI = 0.0–84, and *p* = 0.041).

To study the immediate effect of exposure to TNFi antibodies, sPD-L1 was measured in serum samples of RA patients (Figure 3C) before and 24 h after their first infusion of 200 mg of infliximab. We saw that the sPD-L1 was significantly decreased in 6 of 7 patients after the infliximab infusion (Figure 3D, median of differences = −8.3, 95% CI = −31 to 3.9, and *p* = 0.023). In the 5 patients who left blood samples 2 weeks after the first infusion, 3 patients had elevated levels and 2 patients had suppressed levels of sPD-L1, compared to the pre-infusion sample (**Figure 3D**, not significant). A reduction in sPD-L1 levels was measured in 6 of 7 patients after 2 infusions of infliximab at 6 weeks (Figure 3D, not significant).

### Exposure to Therapeutic TNFα-Inhibiting Antibodies Normalize sPD-L1 Levels in Smokers

We next analyzed sPD-L1 in smoking patients with and without treatment with TNFi. We saw that TNFi reversed the suppressive effect of smoking on sPD-L1 levels. Smokers treated with TNFi had sPD-L1 levels similar to those observed in non-smokers not treated with TNFi (Figure 3B, medians 20 and 16, respectively). The difference in sPD-L1 between the smokers and non-smokers could only be seen if the patients were not treated with TNFi. In the patients not treated with TNFi, smokers had 8 times lower median levels of sPD-L1 compared to non-smokers (Figure 3B, median 2 vs. 16 pg/ml, H–L difference = −6, 95% CI = −12 to 0, and *p* = 0.013).

### Smoking Changes the Fcγ-Receptor Expression Profile and Influences sPD-L1 Shedding

To further explore the mechanism by which antibodies regulated sPD-L1 levels in RA patients, we analyzed the expression of the stimulatory Fc-gamma receptor (FcγR) FcγRIIIA, and of the inhibitory FcγRIIB, in human PBMC.

**Figure 4:**
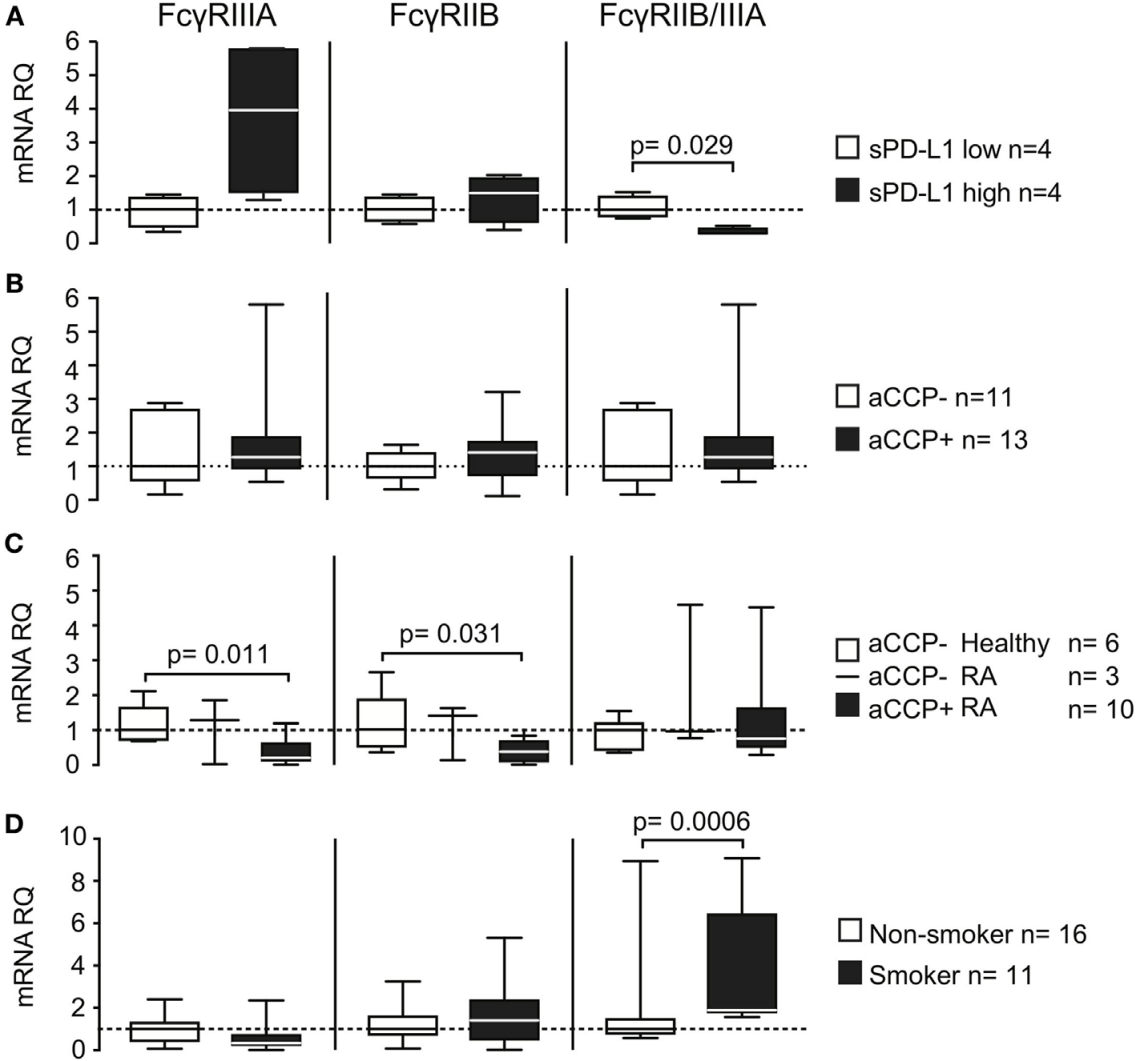
Smoking and anti-cyclic citrullinated peptide (aCCP) antibodies regulate production of programed death protein 1 (PD-L1) through Fcγ-receptors. mRNA levels of Fcγ-receptors (FcγRs) IIIA and IIB and their ratio were measured in (A+B) IgG/LPS stimulated peripheral blood mononuclear cells (PBMC) from aCCP antibody negative healthy controls that produced **(A)** low (<10 pg/ml) or high (>10 pg/ml) levels of PD-L1 in culture; **(B)** stimulated with aCCP+ and aCCP− IgGs; **(C)** T cell depleted PBMC from RA patients and healthy subjects with different aCCP status; **(D)** T cell depleted PBMC from RA patients and healthy subjects with different smoking status. The Mann–Whitney *U* test was applied. Boxes represent median with interquartile range. Whiskers indicate min and max values.

To investigate whether the expression of FcγRs was related to the release of sPD-L1, we compared their expression in IgG-stimulated PBMC cultures. We observed a higher expression of both FcγRIIIA and FcγRIIB mRNA in the cell cultures with higher sPD-L1 (Figure 4A, not significant). FcγRIIIA was increased 4.0 times, while FcγRIIB was increased only 1.5 times. Consequently, the FcγRIIB/FcγRIIIA ratio was significantly lower in the cultures with high sPD-L1 production. This indicated a predominance of the stimulatory FcγRIIIA (median ratio 1.0 vs. 0.30, H–L difference = −0.62, 95% CI = −1.3 to −0.22, and *p* = 0.029).

Direct cell exposure to aCCP + IgG in culture had no significant effect on FcγR (Figure 4B). However, aCCP-positive patients had low expression of both FcγRIIIA (Figure 4C, median RQ 0.21 vs. 1.0, H–L difference = 0.70, 95% CI = 0.35–1.4, and *p* = 0.011) and FcγRIIB (median RQ 0.38 vs. 1.0, H–L difference = 0.63, 95% CI = 0.064–1.6, and *p* = 0.031) compared to aCCP-negative healthy controls.

Finally, we wanted to learn if smoking interfered with the FcγRs expression. For this purpose we analyzed the expression of FcγRs in PBMC enriched with the antigen-presenting cells of 11 smokers and 16 non-smokers. Smokers had somewhat lower expression of FcγRIIIA mRNA, but did not differ from non-smokers in FcγRIIB expression. This resulted in a higher FcγRIIB/FcγRIIIA ratio, characteristic for the inhibitory receptor predominance, in smokers (Figure 4D, median RQ 1.0 vs. 1.9, H–L difference = 1.0, 95% CI = 0.56–2.4, and *p* = 0.00062).

## Discussion

In the present study, we measured the soluble form of PD-L1 in serum of RA patients. We observed that sPD-L1 was connected to systemic inflammation, and its production was suppressed by MTX treatment. Several attempts were previously made to explain why PD-L1 fails to suppress inflammation in RA. It was suggested that the expression of PD-L1 in the RA synovia is sub-optimal to control joint inflammation (19). An alternative explanation is that high expression of PD-1 and PD-L1 in the RA synovia caused a hypo-responsive state in synovial CD4+ T cells (27).

This study revealed an association between smoking and distinctly low levels of serum sPD-L1 in RA patients. We previously reported that the expression of its receptor, PD-1, was limited by smoking (8). Together, these results indicate that smoking may interfere with both sides of the PD-1/PD-L1 system. However, the suppressive effect of smoking on sPD-L1 levels was only observed under condition of inflammation in RA patients. The similar trend to lower sPD-L1 levels in healthy smokers did not reach the level of significance. A previous report indicated similar sPD-L1 levels in smoking and non-smoking lung cancer patients (28). It is possible that the sPD-L1 levels seen in RA patients reflect the expression at the primary site of inflammation. Previous reports state that the PD-L1 expression is induced by the synovia of RA patients and that the synovial level of sPD-L1 is increased (18, 19).

Two aspects need to be considered in the interpretation of low sPD-L1 levels in RA patients. On the one hand, we show that sPD-L1 levels may be low in absence of inflammation, or when inflammation is suppressed by immune modulating treatment. On the other hand, a well-defined function of PD-L1 is to limit the inflammatory response and protect against autoimmunity. Thus, low levels of sPD-L1 could indicate either suppressed inflammation, or insufficient control of the inflammation. To address this issue we conducted a multivariate analysis, which demonstrated that smoking was independently associated with low levels of sPD-L1, and not merely attributed to reduced systemic inflammation. Consequently, smoking is likely to prevent an increase of sPD-L1 levels when protective PD-L1 function is required. We speculate whether the suppression of this essential regulatory system increases the susceptibility to autoimmune reactions and RA. This suggestion is in agreement with the established association between RA and smoking (7).

Anti-CCP antibodies seemingly had a stimulatory effect on sPD-L1 production. However, comparing patients with different disease duration, we observed that aCCP-positive patients in the early phase of the disease did not have higher sPD-L1 levels. We speculate whether the time of exposure to aCCP is of importance for their effect on sPD-L1. Naturally, disease duration is a crude estimation of aCCP exposure, based on the assumption that patients with longer disease duration have been positive for aCCP antibodies for a longer time. Earlier studies indicate that approximately 40% of the patients have aCCP before they develop RA, with a median of 5 years and maximum of 14 years before diagnosis (29). This implies that some patients have been exposed to aCCP for a longer time than indicated by the disease duration. Nevertheless, the aCCP status is generally stable once it has been established (30). The immediate response to aCCP was studied by culturing PBMC in presence of aCCP-positive IgG. The experiment revealed that direct exposure to aCCP limited the release of sPD-L1. We reasoned that the influence of aCCP on sPD-L1 levels was likely mediated through FcγRs. Thus, we hypothesized that therapeutic antibodies might also influence sPD-L1 levels. Indeed, exposure to TNFi generated the pattern of PD-L1 production similar to aCCP. Treatment with infliximab lowered the levels of sPD-L1 in RA patients 24 h after their very first infusion. Lower PD-L1 levels were also seen in patients with shorter disease duration, while higher levels were seen in TNFi-treated patients with disease duration above 7 years. Importantly, we observed that treatment with TNFi reversed the suppressive effect of smoking on sPD-L1 levels. Taken together, these results indicate that immediate exposure to antibodies suppresses sPD-L1 levels, while chronic exposure eventually results in higher production.

We concluded that exposure to the IgG antibodies aCCP and TNFi generated similar effects with regards to sPD-L1. We, therefore, anticipated that the production of sPD-L1 was regulated by FcγRs. Indeed, a predominance of the stimulatory FcγRIIIA was found in PBMCs generating high sPD-L1 levels. PBMC subjected to short-term stimulation with aCCP in culture, and PBMC from patients chronically exposed to aCCP, showed opposing effects on their FcγRs transcription. We speculate that chronic exposure to aCCP influences the expression pattern of FcγRs, thereby explaining the differential effect of aCCP on the sPD-L1 production in these two situations. It was previously shown that aCCP levels in RA patients were associated with the protein expression of FcγRIII (31) and FcγRs of subtypes II and III were up-regulated in the synovia of RA patients (19), demonstrating that expression of these receptors may indeed be disturbed by the presence of aCCP.

Finally, we demonstrated that smokers had a higher ratio of FcγRIIB/-IIIA. We propose that the predominance of the inhibitory FcγRIIB repressed sPD-L1 production, offering an explanation to how smoking and antibodies may interact to influence sPD-L1 levels. Possibly, the infusion of the TNFi antibodies provides enough stimulatory signals to override this misbalance in FcγR expression, and normalized sPD-L1 levels in smokers.

To conclude, the present study shows that serum levels of sPD-L1 in RA are triggered by systemic inflammation. Smoking limits sPD-L1 response in RA smokers by changing the balance in the expression of stimulatory and inhibitory FcγRs. Activation of FcγR by aCCP antibodies and therapeutic TNFi antibodies has differential sPD-L1 production response in the early and late RA, which may mark a switch from acute to chronic inflammation.

## Ethics Statement

This study was carried out in accordance with the recommendations of the Regional Ethical Evaluation Board in Gothenburg, Sweden. The protocol was approved by the Regional Ethical Evaluation Board in Gothenburg. All subjects gave written informed consent in accordance with the Declaration of Helsinki.

## Author Contributions

MB and CW contributed to conception and design of the study. MB, BL, AB, CM, and LE performed clinical evaluation of study participants and collected clinical material. SS recruited patients and organized the database. CW, ME, and RP conducted serological measurements and cell experiments. CW performed the statistical analysis. CW and MB wrote the first draft of the manuscript. All authors contributed to manuscript revision, read and approved the submitted version.

## Conflict of Interest Statement

The authors declare that the research was conducted in the absence of any commercial or financial relationships that could be construed as a potential conflict of interest.

## Footnotes

^1^https://www.ensembl.org/index.html.

^2^http://bioinfo.ut.ee/primer3-0.4.0/.

^3^http://www.premierbiosoft.com/netprimer/.

^4^http://rohsdb.cmb.usc.edu/GBshape/cgi-bin/hgPcr.
